# Group-Based Multitrajectory Modeling Analysis of Joint Trajectories of Blood Pressure and Heart Rate and Their Association With Relative Change in Relative Wall Thickness in Patients With Hypertension: Community-Based Longitudinal Cohort Study

**DOI:** 10.2196/87091

**Published:** 2026-09-09

**Authors:** Qi Pan, Nanxiang Zhang, Shuo Yang, Chonglong Ding, Jianan Yin, Yi Fei, Xinyan Zou, Yongjun Zheng, Xiang Huang, Jiezhen Feng, Hai Lin, Jinxin Zhang

**Affiliations:** 1 Department of Medical Statistics, School of Public Health, Sun Yat-sen University, No. 74, Zhongshan 2nd Road, Yuexiu District, Guangzhou, 510080, China, 2087330678; 2 Sanxiang Community Health Service Center of Zhongshan, Town Goverment of Zhongshan, Zhongshan, China

**Keywords:** hypertension, ambulatory blood pressure monitoring, ventricular wall thickness, cardiac remodeling, group-based multitrajectory modeling

## Abstract

**Background:**

Ambulatory blood pressure monitoring objectively assesses circadian hemodynamic rhythms. However, the joint trajectories of longitudinal blood pressure (BP) and heart rate (HR), and their association with early hypertensive cardiac remodeling, remain incompletely characterized.

**Objective:**

This study aimed to identify joint trajectories of BP and HR using group-based multitrajectory modeling and examine their associations with relative changes in relative wall thickness (*Δ*RWT).

**Methods:**

This community-based longitudinal cohort study included 213 patients with hypertension, with data collected between November 2022 and January 2025. Participants underwent 4 ambulatory BP monitoring sessions over 6 months, and echocardiography was measured at 3 months and 9 months. Group-based multitrajectory modeling was used to identify joint trajectories of 24-hour mean systolic blood pressure, diastolic blood pressure, and HR. A full-model multiple linear regression estimated the associations between trajectory groups and *Δ*RWT. Sensitivity analyses were conducted by adjusting for age and sex, and by using complete-case data.

**Results:**

Four joint trajectory groups were identified: group 1 (lowest BP and HR, n=59), group 2 (normal BP, high HR, n=60), group 3 (stage 1 hypertension range, normal HR, n=54), and group 4 (stage 2 hypertension range, high HR, n=40). In the primary adjusted multiple linear regression model, compared with group 4, group 2 showed a significant inverse association with changes in *Δ*RWT (*b*=−0.059, 95% CI −0.112 to −0.007; *P*=.03). Antidiabetic medication use was significantly associated with a reduction in *Δ*RWT (*b*=−0.067, 95% CI −0.116 to −0.018; *P*=.008). Waking up before 7 AM was positively associated with an increase in *Δ*RWT (*b*=0.045, 95% CI 0.007-0.083; *P*=.02). Notably, the association for group 2 attenuated to marginal significance (*P*=.06) in the complete-case analysis, primarily due to the reduction in statistical power.

**Conclusions:**

Hypertensive cardiac remodeling exhibits significant heterogeneity across distinct joint systolic blood pressure, diastolic blood pressure, and HR trajectories. Stable BP control is associated with a potential attenuation of early cardiac remodeling even in the presence of an elevated HR; however, this beneficial association attenuated to marginal significance in the complete-case analysis. Concurrent antidiabetic medication use and waking after 7 AM showed a beneficial association with the relative change in *Δ*RWT.

## Introduction

Hypertension is a chronic cardiovascular disease (CVD). It involves persistent high arterial blood pressure (BP) and is a top cause of global cardiovascular illness and early death [1,2]. High systolic blood pressure (SBP) was one of the top 3 risk factors for poor health and early death worldwide in 2021, along with smoking and high blood sugar [3]. Its prevalence doubled from 650 million (1990) to 1.3 billion adults (2019) [4].

Ambulatory blood pressure monitoring (ABPM) can objectively and comprehensively assess circadian rhythms and occult hypertension [5,6], and several community-based studies have shown that ambulatory BP is superior to office BP in determining cardiovascular risk [7-9]. Poor BP control raises cardiovascular event and death risks significantly [10]. Hypertension can lead to progressive deterioration of left ventricular (LV) geometry, an important predictor of cardiovascular events [11-13]. Four LV geometric patterns are defined using left ventricular mass index (LVMI) and relative wall thickness (RWT): concentric hypertrophy (CH), eccentric hypertrophy, concentric remodeling, and normal geometry [14]. Left ventricular mass exhibits a continuous association with cardiovascular risk [15]. CH, defined by an elevated LVMI accompanied by RWT, has been shown to be a strong predictor of future cardiovascular events in patients with hypertension [12,16]. RWT serves as a central indicator to distinguish CH from eccentric hypertrophy and directly determines the pathological features of CH [17-19]. Although global wall thickness shows superior discriminatory power in patients with early-stage normal left ventricular mass [20], prognostic studies focusing specifically on RWT remain limited. Given the important role of RWT in LV remodeling patterns and its predictive value for cardiovascular prognosis, this study chooses it as an outcome indicator representing LV geometry.

However, traditional clinical practice relies on a single office BP measurement, which makes it difficult to capture the dynamic changes of BP (ie, BP trajectory) [21]. A single measurement is susceptible to stochastic fluctuation, which may lead to erroneous risk stratification [22,23]. Group-based trajectory modeling identifies the number of subgroups with different developmental trends from longitudinal data and fits the developmental trajectories of different subgroups [24,25]. Extending this framework, group-based multitrajectory modeling (GBMTM) allows researchers to analyze associations among several relevant outcomes over time and across subgroups. In cardiovascular epidemiology, group-based trajectory modeling reveals adverse change patterns in glucose and BP trajectories that predict coronary and cerebrovascular events [26,27]. Recent studies have confirmed that BP trajectories are better predictors of cardiovascular prognosis than a single measurement [28]. However, the relationship between the joint trajectories of BP and heart rate (HR) and cardiac structure in patients with hypertension remains unclear. Previous studies have analyzed SBP, diastolic blood pressure (DBP), and HR as independent variables, overlooking their physiological synergy. However, BP and HR are coregulated by the autonomic nervous system and are often simultaneously influenced by sedatives, analgesics, and antihypertensive drugs [29,30]. Patients with hypertension often exhibit increased resting HR, with more pronounced elevation in those with moderate to severe hypertension [31,32].

Therefore, this study aimed to identify distinct joint trajectories of 24-hour mean SBP, DBP, and HR among community-based patients with hypertension using GBMTM, and to investigate the associations between these trajectory groups and *Δ*RWT, a sensitive marker of early hypertensive cardiac remodeling.

## Methods

### Study Participants

The study cohort (Dynamic ECG & Blood Pressure for Hypertension at Sanxiang) consisted of managed care hypertension cases within the primary care community of Zhongshan City, Guangdong Province, China, from November 1, 2022, to January 1, 2025 [33]. The inclusion criteria for this study were based on meeting the diagnostic criteria for hypertension in the Chinese Hypertension Prevention and Treatment Guidelines (2024 Revised Edition) [34]: SBP ≥140 mm Hg and/or DBP ≥90 mm Hg measured on 3 non–consecutive days within a week. Patients with hypertension who were included in the management of the National Basic Public Health Service Programme were included in the study. Inclusion criteria included baseline age ≥35 years; clear consciousness, unimpeded communication, and no cognitive impairment; and completion of 4 ABPM sessions (at baseline, 3 months, 6 months, and 9 months after baseline; with 3-month intervals). Exclusion criteria were patients with secondary hypertension (such as renovascular hypertension, endocrine-induced hypertension, etc); individuals with gestational hypertension; those complicated with severe acute or chronic diseases (eg, malignant tumors, end-stage renal disease, and acute decompensated heart failure) or severe physical disabilities; those with incomplete baseline data (eg, missing key variables) or inability to cooperate with follow-up; and those who withdrew voluntarily or were lost to follow-up.

### Data Collection

#### Structured Questionnaire

Through reviewing the literature [35-37], a structured, face-to-face questionnaire was administered to collect comprehensive baseline data. This instrument captured sociodemographic characteristics (age, sex, residency, education, and marital status), physical metrics (BMI), lifestyle habits (smoking and alcohol consumption), and current medication use. To specifically evaluate hypertension management, the questionnaire also assessed recent dietary patterns (primarily evaluating the structural balance between meat-based and plant-based diets), history of diabetes (verified via patient self-reporting combined with community electronic health records), and self-reported average morning wake-up time.

For statistical analysis, self-reported morning wake-up times were dichotomized at 7 AM. This specific threshold is grounded in established chronobiological and cardiovascular physiological evidence [38]. During this early morning window, the morning BP surge and cortisol awakening response reach their peak, which corresponds directly to the well-documented surge in major cardiovascular events occurring between 6 AM and 12 PM [39,40]. Physiologically, awakening before 7 AM triggers a premature shift toward sympathetic nervous system dominance and amplifies neuroendocrine stress responses [41,42]; both mechanisms are intrinsically linked to elevated vascular resistance and subsequent adverse cardiac remodeling.

#### Sleep Status Rating Scale

The Self-Rating Scale of Sleep [43] was used to assess the sleep status of the patients. There are 10 items in the scale, and each item is rated on a 5-point scale (1-5), with higher scores indicating more serious sleep problems. Li [43] initially proposed that a total Self-Rating Scale of Sleep score of ≥23 indicates sleep problems.

#### Ambulatory Blood Pressure Monitoring

After completion of baseline data collection, patients with hypertension who completed the baseline assessment received regular follow-up visits. At each visit, we used a certified 24-hour ABPM (Nalon ABP01, Nalon Health Technology Co, Ltd) for 24-hour continuous monitoring (valid readings ≥80%, recorded every 30/60 minutes during daytime or nighttime, respectively), and systematically collected 24-hour mean SBP, DBP, and HR data to comprehensively assess the patients’ BP status.

#### Echocardiography

Echocardiography was performed at 3 months after baseline and 9 months after baseline using a Philips EPIQ CVx ultrasound system with an S5-1 probe (Royal Philips) to objectively and comprehensively obtain the patient’s cardiac-related parameters. RWT was calculated according to the American Society of Echocardiography recommendations [44] as follows: RWT= (2×posterior wall thickness at end-diastole)/left ventricular internal dimension at end-diastole (LVIDd). To strictly control measurement bias, echocardiographic examinations for the same patient were independently performed by a doctor, while the subsequent entry of echocardiographic parameters was handled by 2 separate investigators. Ultimately, all collected data underwent rigorous range and logic checks to ensure database completeness and accuracy.

#### Medication Exposure

Medication exposure during the 9-month follow-up was tracked via prescription refill records, categorizing the predominant regimen into drug classes including angiotensin receptor blockers (ARBs), calcium channel blockers (CCBs), β-blockers, and combination therapies. To account for potential treatment adjustments during this period, each patient’s predominant medication regimen was defined as the specific drug class or combination prescribed most frequently across all outpatient visits.

### Quality Control

To ensure data quality for the managed patients with hypertension, multiple strategies were implemented. All follow-up staff were professionally trained and certified. They strictly adhered to standardized operating procedures during data collection to ensure the consistency and objectivity of raw data.

During data cleaning and validation, abnormal values were defined as clinically implausible data (eg, SBP <80 or >220 mm Hg) or statistical outliers beyond the mean (SD 3). These values were first cross-checked against original records, such as monitoring device logs and paper questionnaires. Confirmed measurement errors were corrected, while unverifiable values were marked as missing.

Missing data were subsequently handled based on variable criticality. Participants missing ≥20% of critical measurements were excluded according to predefined criteria. For noncritical variables and partially missing critical variables (eg, single-time RWT), multiple imputation (MI) was used to generate 5 complete datasets, and the results were pooled for analysis.

To guarantee standardization and accuracy, the processes of data cleaning, merging, and handling missing values were independently repeated twice and cross-compared. Additionally, research variables were classified and assigned values based on high-quality literature. Furthermore, a complete-case analysis (CCA), strictly excluding participants with any missing covariate data, was performed as a sensitivity analysis to verify the robustness of the primary imputed findings.

### Statistical Analyses

#### Modeling of Joint Trajectories of BP and HR

Analysis was performed using SAS (version 9.4; SAS Institute Inc) software, and GBMTM was completed through the SAS Proc Traj program, fitting joint trajectories of the 24-hour mean SBP, DBP, and HR. Models specifying 2-5 trajectory groups, with polynomial orders (constant, linear, and quadratic) for each variable within each group, were evaluated. The optimal number of groups and shape of trajectories were determined based on (1) minimization of Bayesian Information Criterion and Akaike Information Criterion, (2) average posterior probabilities of group membership >0.90 for each group, (3) entropy >0.80, (4) adequate group size (each group >5% of the sample), and (5) clinical interpretability.

#### Association Analysis of Joint Trajectories With *Δ*RWT

Echocardiography was performed at the first and third follow-up visits to obtain cardiac-related parameters of the patients. For missing values of relative ventricular wall thickness measured by echocardiography, 5 complete datasets were generated using MI, and the results were combined and analyzed. The relative change in RWT (*Δ*RWT) over the 6-month period between the first (3 months after baseline) and third (9 months after baseline) follow-up visits was defined as follows: *Δ*RWT was used as the dependent variable, and the data were distributed according to a non–normal distribution. The independent variables included 18 factors such as trajectory group, sociodemographic characteristics (age, gender, residency status, education level, and marital status), physical status (BMI), lifestyle (smoking and alcohol consumption), and treatment factors (medications used). Continuous variables were expressed as median (IQR), and comparisons between groups were made using the Kruskal-Wallis *H* test. Independent variables with *P*<.10 in univariate analyses were simultaneously entered into the regression models using the Enter method. To ensure the robustness of our findings, 2 types of sensitivity analyses were conducted: first, by further adjusting for predefined biological confounders, specifically age and sex; and second, by performing a CCA, which strictly excluded participants with any missing covariate data to verify the consistency of the results. Multicollinearity was diagnosed using the variance inflation factor (VIF), which was considered to exist if the VIF exceeded 4. If multicollinearity was identified, variables with the highest VIF values were sequentially excluded (prioritizing those with weaker clinical relevance to the outcome), until all remaining variables had VIF ≤4, to ensure the stability of the multiple linear regression model. Furthermore, to justify the use of parametric linear regression for the non–normally distributed *Δ*RWT, residual diagnostics were systematically performed. The regression models were deemed statistically appropriate provided that the standardized residuals fell within the widely accepted threshold (approximately ±3), which confirms the absence of severe outliers and ensures that the assumptions of homoscedasticity and normality of residuals were adequately met. The statistical analyses were conducted using R (version 4.4.3; R Foundation for Statistical Computing) and SPSS (version 26.0; IBM Corp).

#### Sensitivity Analyses

To verify the robustness of the primary findings, sensitivity analyses were implemented. The first analysis forced established biological confounders (age and sex) into the regression models to address potential variable selection biases. The second used a CCA approach, strictly excluding any participants with missing covariate data to validate the stability of the imputed associations.

#### Antihypertensive Medications Assessment

Medication exposure during the 9-month follow-up was tracked via prescription refill records, categorizing the predominant regimen into drug classes including ARBs, CCBs, β-blockers, and combination therapies. Differences in medication utilization across the identified trajectory groups were evaluated primarily using chi-square tests.

### Ethical Considerations

The study was approved by the Biomedical Research Ethics Review Committee of the School of Public Health, Sun Yat-sen University (approval number 2021-No.081). Written informed consent was obtained from all participants prior to enrollment. All study data were deidentified before analysis to ensure confidentiality. Participants received complimentary 24-hour ABPM and echocardiography assessments as compensation for their involvement in this research. No images or supplementary materials containing identifiable information are included in this manuscript.

## Results

### Joint Trajectories of 24-Hour Mean SBP, DBP, and HR

The relevant parameters during the fitting trajectory are shown in Table 1. Since the proportion of group members in the group number of 5 was less than 5%, the optimal number of groups for the trajectory model was chosen as 4. Then the number of longitudinal trajectory groups in patients with hypertension was determined to be 4, the order was adjusted, and the order of the final trajectory curve was determined by combining the morphology of the trajectory curves in the graphs and the *P* value, which were all determined to be 0th order (straight lines). All trajectory groups satisfied the following criteria [25]: absolute values of Bayesian Information Criterion and Akaike Information Criterion are minimum, proportion of group members >5%, entropy >0.80, and average posterior probability >0.90, indicating excellent model classification accuracy.

**Table 1.** Model selection criteria for group-based multitrajectory modeling of 24-hour mean blood pressure and heart rate. Data were derived from a longitudinal cohort study conducted at 3 community health centers in Zhongshan City, Guangdong Province, China, among 213 patients with hypertension between November 2022 and January 2025.

Number of groups	BIC^a^	AIC^b^	AvePP^c^	Proportion of group members (%)	Likelihood ratio	Entropy	Number of group members
2	−9443.33	−9406.35	96.59/97.48	52.47/47.53	−9384.35	0.85	110/103
3	−9348.48	−9294.70	93.00/93.53/95.88	27.67/48.99/23.37	−9262.70	0.87	59/105/49
4	−9280.14	−9209.56	96.32/92.45/92.31/94.29	27.90/28.18/25.56/18.36	−9167.56	0.88	59/60/54/40
5	−9247.55	−9160.15	95.30/91.01/93.27/92.14/99.13	26.71/29.73/25.30/14.27/3.99	−9108.15	0.89	57/64/53/31/8

a BIC: Bayesian Information Criterion.

b AIC: Akaike Information Criterion.

c AvePP: average posterior probability.

As described in Table 2, the 4 trajectory groups have the following characteristics: group 1: BP (123/68 mm Hg) and HR (67 bpm) were both at the lowest levels, but a diastolic pressure below 70 mm Hg may lead to inadequate organ perfusion (eg, myocardial ischemia and cerebral ischemia), particularly in older adult patients or those with concomitant coronary artery disease or carotid artery stenosis [45]; group 2: BP was well controlled (128/77 mm Hg), but the persistently elevated HR (80 bpm) suggested increased sympathetic nervous system activity, which may increase cardiovascular risk; group 3: BP was within the stage 1 hypertension range (134/82 mm Hg), with a normal HR (70 bpm); and group 4: BP was within the stage 2 hypertension range (142/89 mm Hg), with high HR (84 bpm), indicating a decompensated state and the highest cardiovascular risk. The median pulse pressure across the 4 study groups ranged from 51 to 55 mm Hg. Although these group-level mean values were below the 60 mm Hg risk threshold specified by the European Society of Hypertension [45], this macro-level observation does not rule out the presence of elevated pulse pressure-related risk in individual patients.

**Table 2.** Baseline blood pressure, heart rate, pulse pressure, and main clinical characteristics of each joint trajectory group. Data were derived from a longitudinal cohort study conducted at 3 community health centers in Zhongshan City, Guangdong Province, China, among 213 patients with hypertension between November 2022 and January 2025.

Group	Group members, n	SBP^a^, mm Hg	DBP^b^, mm Hg	Pulse pressure, mm Hg	HR^c^, bpm	Main characteristics
1	59	123	68	55	67	Lowest BP^d^ and HR
2	60	128	77	51	80	Normal BP and high HR
3	54	134	82	52	70	Stage 1 hypertension range, normal HR
4	40	142	89	53	84	Stage 2 hypertension range, high HR

a SBP: systolic blood pressure.

b DBP: diastolic blood pressure.

c HR: heart rate.

d BP: blood pressure.

**Figure 1.**
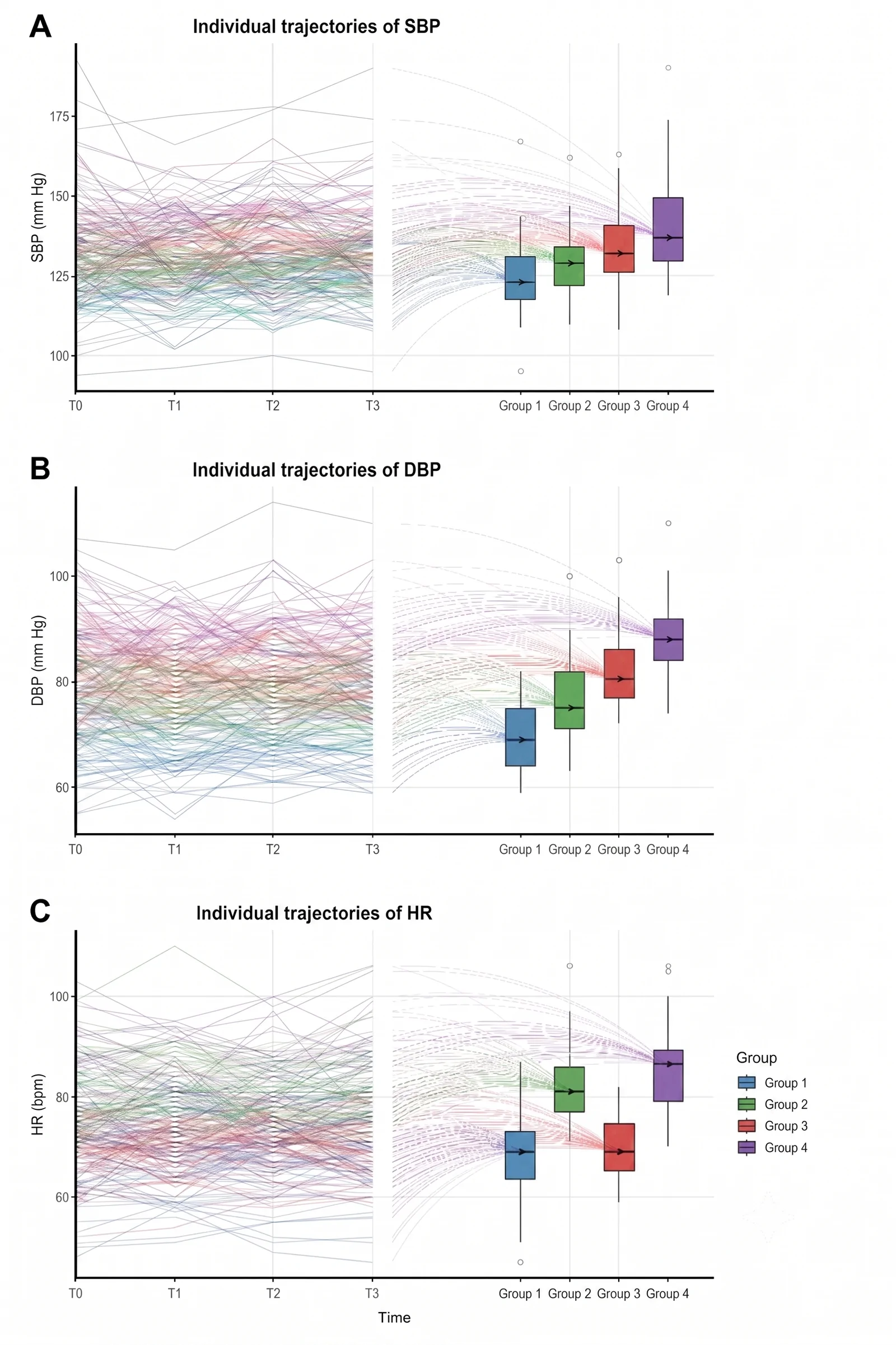
Individual longitudinal trajectories of 24-hour mean SBP, DBP, and HR. The spaghetti plots depict the individual hemodynamic progression of patients across the 4 identified trajectory groups (groups 1-4) over 6 months, with measurements taken at baseline (T0), 3 months (T1), 6 months (T2), and 9 months (T3). Data were derived from a longitudinal cohort study conducted at 3 community health centers in Zhongshan City, Guangdong Province, China, among 213 patients with hypertension between November 2022 and January 2025. DBP: diastolic blood pressure; HR: heart rate; SBP: systolic blood pressure.

**Figure 2.**
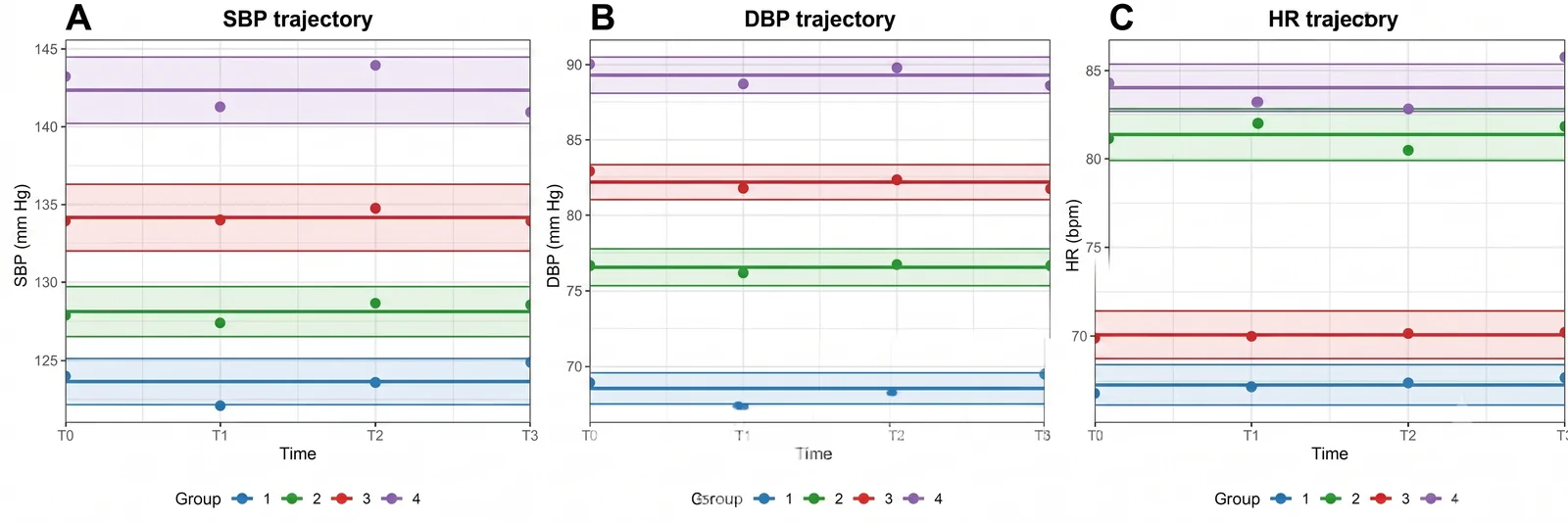
Joint trajectories of 24-hour mean SBP, DBP, and HR identified by group-based multitrajectory modeling (GBMTM). The line graphs illustrate the 4 distinct joint trajectory groups over 4 time points: baseline (T0), 3 months (**T1**), 6 months (T2), and 9 months (T3). Shaded bands around each trajectory represent the 95% CIs of the GBMTM-predicted values. Data were derived from a longitudinal cohort study conducted at 3 community health centers in Zhongshan City, Guangdong Province, China, among 213 patients with hypertension between November 2022 and January 2025. DBP: diastolic blood pressure; HR: heart rate; SBP: systolic blood pressure.

**Figure 3.**
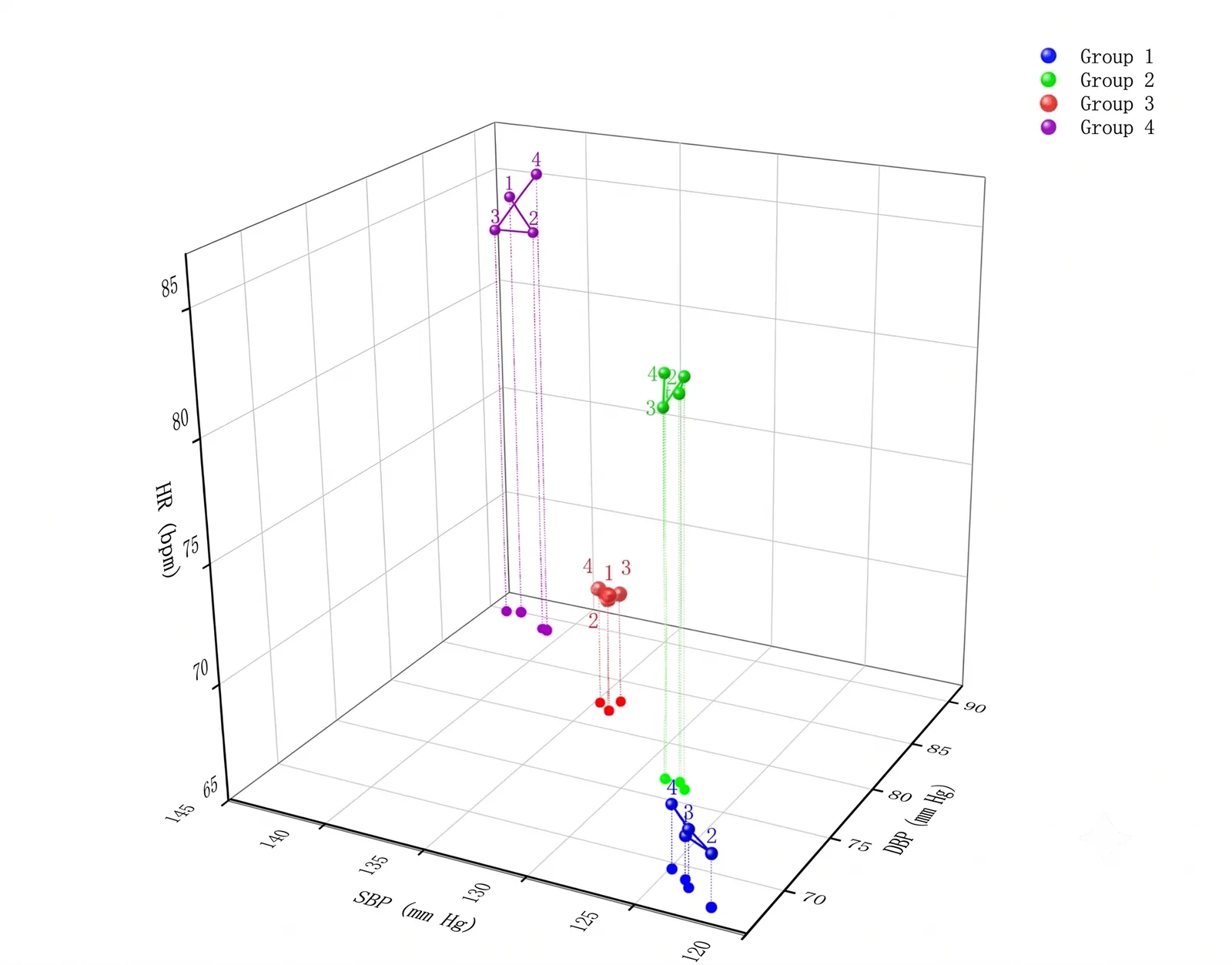
Three-dimensional (3D) spatiotemporal distribution of actual mean values for joint trajectories of SBP, DBP, and HR. The 3D scatter plots quantitatively distinguish the spatiotemporal differentiation among the 4 trajectory groups across 4 follow-up visits: baseline (1), 3 months (2), 6 months (3), and 9 months (4). Data were derived from a longitudinal cohort study conducted at 3 community health centers in Zhongshan City, Guangdong Province, China, among 213 patients with hypertension between November 2022 and January 2025. DBP: diastolic blood pressure; HR: heart rate; SBP: systolic blood pressure.

The individual trajectories of BP and HR in 213 patients with hypertension (Figures 1A-1C) showed strong alignment with the joint trajectories identified by the GBMTM (Figures 2A-2C). Moreover, the empirical distributions of BP and HR at 9 months after baseline closely matched the model-predicted values for each trajectory group, confirming that the GBMTM reliably identified subgroups exhibiting distinct hemodynamic progression patterns. As illustrated in Figure 3, the 3D distribution of joint trajectories quantitatively distinguishes the spatiotemporal differentiation among the 4 trajectory groups within the multidimensional parameter space. This representation offers clinically valuable insights into the heterogeneous dynamic profiles across these physiological dimensions.

### Baseline Characteristics

The baseline characteristics of the study population are shown in Table 3. A total of 213 patients with hypertension were included in the study, 110 (51.64%) males and 103 (48.36%) females; their age ranged from 35 to 80 years, with a median age of 57 (IQR 52‐65) years. The results of the Kruskal-Wallis *H* test indicated that the 4 factors of trajectory group, wake-up time, dietary pattern, and history of diabetes mellitus met the prespecified threshold for inclusion in the multivariable model (*P*<.10).

**Table 3.** Baseline sociodemographic and clinical characteristics of the study population. Data were derived from a longitudinal cohort study conducted at 3 community health centers in Zhongshan City, Guangdong Province, China, among 213 patients with hypertension between November 2022 and January 2025^a^.

Variable	Values, n	Relative change in RWT^b^ (*Δ*RWT), median (IQR)	*H*	*P* value
Group			8.07	.05
1	59	0.02 (−0.03 to 0.07)		
2	60	−0.05 (−0.10 to 0.03)		
3	54	0.00 (−0.07 to 0.08)		
4	40	0.03 (−0.06 to 0.13)		
Sex			0.01	.92
Male	110	0.00 (−0.08 to 0.08)		
Female	103	0.00 (−0.06 to 0.06)		
Age (years)			2.12	.35
35-44	9	0.00 (−0.03 to 0.01)		
45-59	113	0.00 (−0.07 to 0.09)		
≥60	91	−0.02 (−0.08 to 0.05)		
Registered residence			0.08	.78
Local resident	57	−0.01 (−0.07 to 0.05)		
Non–local resident	156	0.00 (−0.07 to 0.08)		
Education level			0.59	.74
Primary school and below	79	0.00 (−0.07 to 0.05)		
Junior high school	106	0.00 (−0.07 to 0.08)		
High school and above	28	0.00 (−0.10 to 0.07)		
Employment status			0.08	.78
Full-time or part-time	108	0.00 (−0.07 to 0.08)		
Retired or unemployed	105	0.00 (−0.07 to 0.06)		
Marital status			0.10	.75
Married	207	0.00 (−0.07 to 0.08)		
Nonmarried	6	−0.01 (−0.06 to 0.04)		
Monthly household income (RMB)^c^			0.51	.78
<5000	126	0.00 (−0.07 to 0.07)		
5000-9999	61	0.00 (−0.07 to 0.07)		
≥10,000	26	0.00 (−0.06 to 0.08)		
BMI			1.86	.60
Underweight	2	−0.06 (−0.07 to −0.06)		
Normal	72	0.00 (−0.07 to 0.09)		
Overweight	95	−0.02 (−0.07 to 0.07)		
Obese	44	0.01 (−0.05 to 0.07)		
Smoking status			0.69	.71
Smoking	25	−0.02 (−0.07 to 0.08)		
Quit smoking	12	−0.03 (−0.09 to 0.03)		
Never smoked	176	0.00 (−0.07 to 0.07)		
Alcohol consumption			0.00	.99
Regular drinking (≥1/wk)	27	0.00 (−0.08 to 0.08)		
Less frequent drinking (<1/wk)	186	0.00 (−0.07 to 0.07)		
Wake-up time			3.04	.08
Before 7 AM	139	0.00 (−0.07 to 0.09)		
After 7 AM	74	−0.02 (−0.08 to 0.05)		
Bedtime			0.21	.65
Before 10 PM	48	0.00 (−0.08 to 0.12)		
After 10 PM	165	0.00 (−0.07 to 0.07)		
Sleep problems			0.01	.92
Yes	75	0.02 (−0.09 to 0.07)		
No	138	0.00 (−0.07 to 0.07)		
Dietary pattern			4.97	.08
Balanced diet	178	0.00 (−0.07 to 0.08)		
Meat-based diet	17	0.05 (−0.02 to 0.09)		
Plant-based diet	18	−0.05 (−0.13 to 0.03)		
History of diabetes			7.55	.02
Diabetes without medications	7	0.09 (−0.07 to 0.18)		
Diabetes with medications	33	−0.05 (−0.13 to 0.03)		
No diabetes	173	0.00 (−0.07 to 0.08)		
Antihypertensive medications			2.01	.57
ARB^d^	38	−0.03 (−0.09 to 0.05)		
CCB^e^	61	0.00 (−0.07 to 0.08)		
β-blocker	2	0.03 (0.03 to 0.03)		
Combination therapy	70	0.00 (−0.06 to 0.10)		
Sedentary behavior			1.01	.31
Yes	146	0.00 (−0.08 to 0.08)		
No	67	0.00 (−0.07 to 0.05)		

a The data for antihypertensive medication usage are based on available records (N=171). A total of 42 patients with unrecorded or missing medication data were excluded from this specific analysis.

b RWT: relative wall thickness.

c All household income values are presented in Chinese Yuan (RMB). The exchange rate was 1 RMB=0.138 US $ as of January 2025 (the study period).

d ARB: angiotensin receptor blocker.

e CCB: calcium channel blocker.

### Multiple Linear Regression of *Δ*RWT

A multiple linear regression analysis was performed to model *Δ*RWT using the independent variables identified via univariate analysis. The corresponding results are presented in Table 4. Trajectory group, wake-up time, dietary pattern, and history of diabetes were converted to dummy variables, with group 4, wake-up time after 7 AM, balanced diet, and no diabetes set as reference categories. Multicollinearity diagnostics showed all VIFs <2.0.

**Table 4.** Multiple linear regression analysis of the independent associations of joint blood pressure and heart rate trajectories and baseline clinical covariates with the relative change in relative wall thickness (*Δ*RWT). Data were derived from a longitudinal cohort study conducted at 3 community health centers in Zhongshan City, Guangdong Province, China, among 213 patients with hypertension between November 2022 and January 2025^a^.

Variable	*b* ^b^	*b*’^c^	SE	*t *test (*df*)	*P* value	95% CI of *b*
Group						
1	−0.016	−0.056	0.027	−0.598 (204)	.55	−0.070 to 0.037
2	−0.059	−0.205	0.027	−2.224 (204)	.03	−0.112 to −0.007
3	−0.008	−0.027	0.028	−0.292 (204)	.77	−0.063 to 0.047
4	Reference	N/A^d^	N/A	N/A	N/A	N/A
History of diabetes						
No diabetes	Reference	N/A	N/A	N/A	N/A	N/A
Diabetes without medications	0.042	0.060	0.049	0.845 (204)	.40	−0.056 to 0.139
Diabetes with medications	−0.067	−0.188	0.025	−2.681 (204)	.008	−0.116 to −0.018
Wake-up time						
After 7 AM	Reference	N/A	N/A	N/A	N/A	N/A
Before 7 AM	0.045	0.167	0.019	2.354 (204)	.02	0.007 to 0.083
Dietary pattern						
Balanced diet	Reference	N/A	N/A	N/A	N/A	N/A
Meat-based diet	0.054	0.116	0.032	1.663 (204)	.10	−0.010 to 0.117
Plant-based diet	−0.049	−0.107	0.032	−1.542 (204)	.13	−0.112 to 0.014

a *F*=3.392, *P*=.001, *R²*=0.127, adjusted *R²*=0.089. Model diagnostics: the assumption of normally distributed residuals was confirmed. The standardized residuals for this fully adjusted model ranged from −2.127 to 3.001, indicating the absence of severe outliers and justifying the use of parametric multiple linear regression for the non–normally distributed *Δ*RWT.

b *b*: unstandardized coefficient.

c *b’*: standardized coefficient.

d N/A: not applicable.

Results indicated that trajectory group 2 (*b*=−0.059, 95% CI −0.112 to −0.007) and diabetes with medications (*b*=−0.067, 95% CI −0.116 to −0.018) were independently associated with a reduction in *Δ*RWT. Conversely, a wake-up time before 7 AM (*b*=0.045, 95% CI 0.007-0.083) was associated with an increase in *Δ*RWT (Table 4). The fully adjusted multiple linear regression model demonstrated a significant overall fit (*F*_8204_=3.392; *P*=.001), explaining 12.7% of the variance in *Δ*RWT (*R*²=0.127, adjusted *R*²=0.089). This indicates that group 2, medication history, and wake-up time are independently associated with *Δ*RWT, although the overall variance explained by the model remains modest.

### Sensitivity Analysis

To address potential variable selection and imputation biases, sensitivity analyses were performed by forcing age and sex into the regression models. In the sensitivity analysis using the fully adjusted model (Table 5), the association for group 2 remained statistically significant (*P*=.04). Furthermore, antidiabetic medication use (*P*=.009) and waking before 7 AM (*P*=.01) remained independently associated with *Δ*RWT. In this imputed model, age (*P*=.09) and sex (*P*=.66) did not reach statistical significance.

**Table 5.** Sensitivity analysis: fully adjusted multiple linear regression model for the association with change in relative wall thickness (*Δ*RWT). Sensitivity analysis forcing established biological confounders (age and sex) into the multiple linear regression model. Data were derived from a longitudinal cohort study conducted at 3 community health centers in Zhongshan City, Guangdong Province, China, among 213 patients with hypertension between November 2022 and January 2025^a^.

Variable	*b^b^*	*b*’^c^	SE	*t *test (*df*)	*P* value	95% CI of *b*
Group						
1	−0.002	−0.008	0.029	−0.081 (202)	.94	−0.059 to 0.054
2	−0.056	−0.194	0.027	−2.082 (202)	.04	−0.109 to −0.003
3	−0.002	−0.008	0.028	−0.088 (202)	.93	−0.057 to 0.052
4	Reference	N/A^d^	N/A	N/A	N/A	N/A
History of diabetes						
No diabetes	Reference	N/A	N/A	N/A	N/A	N/A
Diabetes without medications	0.039	0.056	0.050	0.783 (202)	.44	−0.059 to 0.137
Diabetes with medications	−0.067	−0.187	0.025	−2.639 (202)	.009	−0.116 to −0.017
Wake-up time						
After 7 AM	Reference	N/A	N/A	N/A	N/A	N/A
Before 7 AM	0.051	0.188	0.019	2.606 (202)	.01	0.012 to 0.089
Dietary pattern						
Balanced diet	Reference	N/A	N/A	N/A	N/A	N/A
Meat-based diet	0.056	0.121	0.032	1.725 (202)	.09	−0.008 to 0.119
Plant-based diet	−0.047	−0.102	0.032	−1.484 (202)	.14	−0.110 to 0.016
Age (years)	−0.002	−0.129	0.001	−1.731 (202)	.09	−0.004 to 0.000
Sex						
Female	Reference	N/A	N/A	N/A	N/A	N/A
Male	−0.008	−0.032	0.019	−0.439 (202)	.66	−0.045 to 0.029

a *F*=3.045, *P*=.001, *R²*= 0.141, adjusted *R²*= 0.095. Model diagnostics: the assumption of normally distributed residuals was confirmed. The standardized residuals for this fully adjusted model ranged from −2.084 to 3.105, indicating the absence of severe outliers and justifying the use of parametric multiple linear regression for the non–normally distributed *Δ*RWT.

b *b*: unstandardized coefficient.

c *b’:* standardized coefficient.

d N/A: not applicable.

To further verify the robustness of these findings, a CCA was conducted (Table 6). In this model, the association for group 2 was attenuated to marginal significance (*P*=.06). This shift is primarily attributable to the reduction in statistical power when cases with missing data were excluded (n=196). Meanwhile, early waking (*P*=.004) and antidiabetic medication use (*P*=.01) robustly maintained their statistical associations. Furthermore, age emerged as an independent factor associated with *Δ*RWT (*P*=.047), whereas sex remained nonsignificant (*P*=.82).

**Table 6.** Sensitivity analysis: fully adjusted multiple linear regression model (complete case analysis) sensitivity analysis excluding missing data and forcing established biological confounders (age and sex) into the model. Data were derived from a longitudinal cohort study conducted at 3 community health centers in Zhongshan City, Guangdong Province, China, among 213 patients with hypertension between November 2022 and January 2025^a^.

Variable	*b* ^b^	*b*’^c^	SE	*t* test (*df*)	*P* value	95% CI of *b*
Group						
1	0.003	0.012	0.027	0.123 (185)	.90	−0.050 to 0.056
2	−0.048	−0.170	0.025	−1.908 (185)	.06	−0.097 to 0.002
3	−0.006	−0.022	0.026	−0.246 (185)	.81	−0.058 to 0.045
4	Reference	N/A^d^	N/A	N/A	N/A	N/A
History of diabetes						
No diabetes	Reference	N/A	N/A	N/A	N/A	N/A
Diabetes without medications	0.018	0.070	0.024	0.753 (185)	.45	−0.029 to 0.066
Diabetes with medications	−0.081	−0.231	0.032	−2.495 (185)	.01	−0.144 to −0.017
Wake-up time						
After 7 AM	Reference	N/A	N/A	N/A	N/A	N/A
Before 7 AM	0.053	0.199	0.018	2.897	.004	0.017 to 0.089
Dietary pattern						
Balanced diet	Reference	N/A	N/A	N/A	N/A	N/A
Meat-based diet	0.055	0.119	0.031	1.768 (185)	.08	−0.006 to 0.117
Plant-based diet	−0.045	−0.100	0.030	−1.506 (185)	.13	−0.104 to 0.014
Age (years)	−0.002	−0.143	0.001	−2.001 (185)	.047	−0.004 to −0.0003
Sex						
Female	Reference	N/A	N/A	N/A	N/A	N/A
Male	−0.004	−0.016	0.017	−0.228 (185)	.82	−0.038 to 0.030

a *F*=3.090, *P*=.001, *R²*= 0.133, adjusted *R²*=0.090. Model diagnostics: the assumption of normally distributed residuals was confirmed. The standardized residuals for this fully adjusted model ranged from –2.301 to 2.939, indicating the absence of severe outliers and justifying the use of parametric multiple linear regression for the non–normally distributed *Δ*RWT. This table presents the results of the complete-case analysis, excluding 17 participants with missing echocardiographic data (final analytical sample size, N=196).

b *b*: unstandardized coefficient.

c *b’:* standardized coefficient.

d N/A: not applicable.

### Antihypertensive Medications Analysis

To investigate differences in the use of antihypertensive medications across trajectory groups, this study categorized patients into 5 groups based on their medication regimens over a 9-month period: ARBs, CCBs, β-blockers, combination therapy (≥2 medications), and unrecorded medications. Given the elevated HR in group 2 and the potential chronotropic effects of CCBs, we focused on comparing CCB usage rates across the 4 trajectory groups. Chi-square analysis revealed no statistically significant differences in overall antihypertensive drug distribution (*χ*²_9_=8.22; *P*=.51) or CCB usage rates (*χ*²_3_=3.30; *P*=.35) across groups (Table 7). Notably, group 4 demonstrated the highest CCB adoption rate (85.7%, predominantly amlodipine), exceeding group 2’s rate (78.7%) by 7.0 percentage points, although this difference did not reach statistical significance. Amlodipine is primarily indicated for patients requiring BP reduction without sympathetic activation or metabolic abnormalities, and its HR-lowering effect is weaker than that of specific CCBs such as cilnidipine [46]. Furthermore, the VALUE trial demonstrated that amlodipine may induce reflex sympathetic activation via peripheral vasodilation, leading to a slight HR increase [47]. While this mechanism supports BP stability, it compromises effective HR control. The prevalent use of amlodipine in Zhongshan’s 3 communities may contribute to sustained HR ≥80 bpm in these patients.

**Table 7.** Chi-square analysis of antihypertensive medication usage across the 4 joint trajectory groups over a 9-month period. Data were derived from a longitudinal cohort study conducted at 3 community health centers in Zhongshan City, Guangdong Province, China, among 213 patients with hypertension between November 2022 and January 2025^a^.

Medications used	Group 1	Group 2	Group 3	Group 4	Chi-square (*df*)	*P* value
Antihypertensive medications	N/A^b^	N/A	N/A	N/A	8.22 (9)	.51
ARB^c^	12	11	11	4	N/A	N/A
CCB^d^	14	18	18	11	N/A	N/A
β-blocker	1	1	0	0	N/A	N/A
Combination therapy	18	18	14	20	N/A	N/A
CCB adoption	N/A	N/A	N/A	N/A	3.30 (3)	.35
Yes	31	37	33	30	N/A	N/A
No	14	10	11	5	N/A	N/A

a The data for antihypertensive medication usage are based on available records (N=171). A total of 42 patients with unrecorded or missing medication data were excluded from this specific analysis.

b N/A: not applicable.

c ARB: angiotensin receptor blocker.

d CCB: calcium channel blocker.

## Discussion

### Principal Findings

In this community-based cohort, GBMTM identified 4 distinct joint trajectory groups of BP and HR. Regarding the clinical implications, group 2 (characterized by normal BP and high HR) was significantly associated with a reduction in *Δ*RWT over 6 months, suggesting a potential improvement in LV geometry. BP in this group was well controlled and below the current guideline-recommended standard for hypertension of 130/80 mm Hg [45,48,49].

Furthermore, the use of antidiabetic medications in patients with hypertension with comorbid diabetes was significantly associated with a reduction in *Δ*RWT. This suggests that ameliorating insulin resistance may inhibit myocardial fibrosis and facilitate the repair of microvascular dysfunction [50-52], thereby providing evidence for the potential beneficial structural associations of these medications. Conversely, a wake-up time before 7 AM was significantly associated with an increase in *Δ*RWT, indicating that circadian dysregulation (manifested as early awakening) may elevate the risk of hypertensive cardiac remodeling by disrupting metabolic, endocrine, and BP regulation [42,53,54].

### Comparison With Prior Work

In this study, unlike previous studies focusing solely on BP trajectories, we applied GBMTM to identify 4 distinct joint trajectories of 24-hour mean SBP, DBP, and HR in patients with hypertension. Previous studies have used the product of BP and HR (BP × HR), known as the double product, as an indicator of myocardial oxygen consumption. The double product serves as an indicator that simultaneously integrates BP and HR. It not only reflects the risk of angina pectoris episodes but also independently predicts all-cause mortality and cardiovascular mortality at rest, highlighting the necessity of simultaneously monitoring BP and HR in clinical assessment and risk prediction [55,56]. This metric increases with age in untreated populations and shows a U-shaped trend in treated populations. The dynamic changes of this index suggest that the synergistic elevation of BP and HR increases myocardial oxygen consumption, while their combined control may reduce related risks. This indicates that there is still significant room for improvement if the focus is solely on BP control, and combined management with other indicators (such as HR) may enhance control effectiveness [57].

Group 1 exhibited the lowest BP and HR levels; however, their DBP fell below 70 mm Hg. Consistent with the “J-curve” phenomenon in cardiovascular risk, clinical management for this phenotype should prioritize systolic BP control while closely monitoring DBP to avoid potential hypoperfusion [58].

Group 2 maintained optimal BP control (below the 130/80 mm Hg threshold) alongside improved ventricular structure, which aligns with findings that good BP control is associated with the potential reversal of LV hypertrophy [59]. However, their elevated resting HR (approximately 80 bpm) suggests persistent sympathetic activation. The structural improvement in *Δ*RWT in these patients may have preceded the reduction in HR, similar to the phenomenon of delayed autonomic modulation (HR variability) after structural improvement observed in patients with chronic thromboembolic pulmonary hypertension [60]. Despite this short-term structural improvement, long-term high HR may still pose potential risks to myocardial tissue, warranting caution. This study, through GBMTM, is the first attempt to reveal the cardiac remodeling characteristics of normal BP with high HR, providing a new perspective for risk stratification in patients with hypertension. These findings suggest that neglecting HR may lead to an underestimation of the potential beneficial associations with cardiac geometry in some patients. Therefore, a combined assessment of BP and HR is necessary for evaluating risk in patients with hypertension.

Group 3 and group 4 represent escalating stages of pressure overload and autonomic decompensation, requiring aggressive, combined targeted therapies. Previous studies have shown that BP trajectories, by integrating long-term BP levels and the slope of changes, can predict cardiovascular events and mortality, providing important evidence for the risk assessment and intervention of CVDs. SBP trajectories have a stronger association with cardiovascular outcomes; furthermore, high-increasing SBP trajectories are also significantly correlated with elevated LVMI [61,62]. Previous studies have confirmed that elevated RWT is an independent predictor of CVD in patients with hypertension and diabetes [63]. This study reveals the synergistic association of dynamic interaction patterns between BP and HR with cardiac remodeling. Furthermore, regarding sleep patterns, the observed risk associated with early awakening aligns with chronobiological evidence that the morning BP surge and cortisol awakening response peak during the early morning window. Awakening prematurely triggers a shift toward sympathetic dominance and amplifies neuroendocrine stress, which are mechanistically linked to elevated vascular resistance. Finally, the observed potential beneficial associations of antidiabetic medications suggest that ameliorating insulin resistance may be linked to the inhibition of myocardial fibrosis and the improvement of microvascular dysfunction.

Our findings also highlight the clinical implication of 0th-order trajectories. The GBMTM algorithm identified 0th-order trajectories as the optimal fit for all 4 groups, indicating that the joint BP and HR profiles remained stable over the 9-month follow-up. This stability aligns with the clinical characteristics of the study cohort, which consisted of patients with chronic hypertension maintained on established medication regimens in a community setting, rather than newly diagnosed patients undergoing initial dose titration. Consequently, the 0th-order trajectories demonstrate that the identified hemodynamic phenotypes, such as the persistently elevated HR in group 2, represent chronic physiological states rather than transient measurements. Establishing this longitudinal stability provides the necessary methodological basis for assessing the cumulative longitudinal associations of these sustained profiles with continuous cardiac structural remodeling.

### Strengths and Limitations

Our study possesses several key strengths. First, methodologically, this study applies GBMTM to a community-based hypertensive cohort to construct joint trajectories of SBP, DBP, and HR. This approach overcomes the limitations of traditional single-variable trajectory analyses, capturing the population heterogeneity of joint hemodynamic changes and identifying subgroups with distinct pathophysiological profiles. Second, by evaluating the progression of early cardiac remodeling across these joint trajectories, we explored the longitudinal determinants of structural changes. In contrast to previous studies that predominantly relied on cross-sectional associations, demonstrating the link between specific trajectory phenotypes and cardiac remodeling progression deepens our understanding of BP-HR interactions and provides longitudinal evidence for early intervention. Third, based on a Chinese community-dwelling cohort, this study highlights the optimal BP control range, the critical need for concurrent HR management, and the clinical significance of sleep interventions. These findings support the implementation of more precise, individualized hypertension management and cardiovascular risk reduction strategies within primary care settings.

However, several limitations of this study must be acknowledged. First, selection bias cannot be completely ruled out, as the sample was derived from a single community. Second, the specific roles of different classes of antidiabetic medications were not explored. Third, the follow-up period was relatively short. Although this duration was sufficient to observe early changes in *Δ*RWT, it is inadequate to fully evaluate long-term target organ damage and hard clinical end points. Fourth, echocardiographic measurements were obtained at the 3-month and 9-month follow-ups, rather than at baseline. Baseline imaging was omitted to reduce the logistical burden on primary care facilities during initial community recruitment. Consequently, the first 3 months functioned as a run-in period, during which approximately 200 participants were lost to follow-up. This attrition may have introduced selection bias, restricting the final cohort to patients with higher adherence. Furthermore, the shortened 6-month observation window (from month 3 to month 9) restricted the detectable magnitude of changes in *Δ*RWT. Because cardiac structural remodeling progresses slowly, this reduced time frame increases the likelihood that true biological changes are masked by the inherent measurement error of echocardiography, which may have attenuated the observed associations in our analysis. Nonetheless, the fact that significant correlations were identified despite these constraints suggests that the associations between high HR, BP trajectories, and cardiac remodeling are clinically noteworthy and warrant attention even within a limited observation window. Fifth, the utilization rate of β-blockers within our cohort was extremely low. Because β-blockers are the primary pharmacological agents for HR reduction, their underuse in this community setting likely contributed to the prevalence of the “normal BP, high HR” phenotype observed in group 2. Consequently, our findings primarily reflect the hemodynamic progression of patients with hypertension lacking specific HR-targeted interventions. This restricts the generalizability of our results to advanced clinical settings where β-blocker prescription rates are substantially higher. Sixth, regarding medication adjustments, we characterized each patient’s pharmacological exposure based on their most frequently prescribed regimen over the 9-month period. While this “predominant exposure” approach effectively captures the primary long-term maintenance therapy typical of community management, it inevitably simplifies the longitudinal pharmacological history. Transient dose titrations or brief medication switches were not continuously modeled as time-varying covariates in the GBMTM. Therefore, the acute hemodynamic impacts of short-term prescription fluctuations cannot be entirely ruled out. Seventh, the relatively modest sample size of our community cohort renders the multiple linear regression models somewhat sensitive to specific data-handling techniques. This is evidenced by the minor discrepancies observed between the sensitivity analysis using imputed data and the strict CCA. Specifically, the association for group 2 attenuated from statistical significance to marginal significance in the CCA. This shift is primarily attributable to the reduction in statistical power when cases with missing data were excluded. Conversely, age emerged as an independently associated factor in the CCA but did not reach full statistical significance in the imputed model. This suggests that in a limited sample, the additional variance introduced by the MI process may dilute certain subtle associations, whereas complete-case exclusion may slightly alter the overall sample composition. These fluctuations highlight the necessity of interpreting the statistical associations cautiously and underscore the need for larger cohort studies to confirm these findings.

### Future Directions

Future large-scale, multicenter prospective cohort studies with extended follow-up periods are needed to fully evaluate long-term cardiac target organ damage and hard clinical end points. Therefore, future research should extend the follow-up period beyond 2 years to better elucidate the temporal relationship between HR optimization and structural improvements, particularly for patients in group 2. Expanding recruitment across diverse community populations will help verify the external validity and generalizability of our findings. Additionally, using objective sleep measurement tools, such as polysomnography, would minimize self-reporting bias and improve the accuracy of sleep parameter assessments.

### Conclusions

In this study, we applied GBMTM to longitudinal data on SBP, DBP, and HR in community-dwelling patients with hypertension, identifying 4 distinct joint trajectory groups of BP and HR. Notably, group 2 (characterized by normal BP and high HR) was significantly associated with a reduction in *Δ*RWT over 6 months, suggesting that achieving stable BP control is linked to a potential short-term attenuation of adverse LV geometric remodeling. However, it should be noted that this beneficial association attenuated to marginal significance in the CCA due to the reduction in statistical power. Additionally, concurrent antidiabetic medication use in patients with hypertension with comorbid diabetes was significantly associated with a reduction in *Δ*RWT, further supporting that these medications showed a beneficial association with the relative change in RWT. Conversely, waking up before 7 AM was associated with an increase in *Δ*RWT (Table 4), indicating that circadian rhythm disruption is involved in the progression of hypertensive cardiac remodeling.

These findings suggest the necessity to incorporate multidimensional BP and HR metrics to refine risk stratification and cardiac management in hypertension. Targeted interventions need to be explored in the future through long-term follow-up and the integration of multidimensional cardiovascular measurements.

## Abbreviations

ABPM: ambulatory blood pressure monitoring
ARB: angiotensin receptor blocker
BP: blood pressure
CCA: complete-case analysis
CCB: calcium channel blocker
CH: concentric hypertrophy
CVD: cardiovascular disease
DBP: diastolic blood pressure
GBMTM: group-based multitrajectory modeling
HR: heart rate
LV: left ventricular
LVIDd: left ventricular internal dimension at end-diastole
LVMI: ventricular mass index
MI: multiple imputation
RWT: relative wall thickness
SBP: systolic blood pressure
VIF: variance inflation factor
ΔRWT: changes in relative wall thickness

## References

[1] Mills KT, Stefanescu A, He J (2020). The global epidemiology of hypertension. Nat Rev Nephrol.

[2] Mills KT, Bundy JD, Kelly TN (2016). Global disparities of hypertension prevalence and control: a systematic analysis of population-based studies from 90 countries. Circulation.

[3] Global burden of disease 2021: findings from the GBD 2021 study. Institute for Health Metrics and Evaluation.

[4] Global report on hypertension: the race against a silent killer. World Health Organization.

[5] Pickering TG, Shimbo D, Haas D (2006). Ambulatory blood-pressure monitoring. N Engl J Med.

[6] Kikuya M, Ohkubo T, Asayama K (2005). Ambulatory blood pressure and 10-year risk of cardiovascular and noncardiovascular mortality: the Ohasama study. Hypertension.

[7] Dolan E, Stanton A, Thijs L (2005). Superiority of ambulatory over clinic blood pressure measurement in predicting mortality: the Dublin outcome study. Hypertension.

[8] Gasowski J, Li Y, Kuznetsova T (2008). Is “usual” blood pressure a proxy for 24-h ambulatory blood pressure in predicting cardiovascular outcomes?. Am J Hypertens.

[9] Hollenberg NK (2004). Prognostic value of ambulatory blood pressure recordings in patients with treated hypertension. Curr Hypertens Rep.

[10] Rothwell PM, Howard SC, Dolan E (2010). Prognostic significance of visit-to-visit variability, maximum systolic blood pressure, and episodic hypertension. Lancet.

[11] Levy D, Garrison RJ, Savage DD, Kannel WB, Castelli WP (1990). Prognostic implications of echocardiographically determined left ventricular mass in the Framingham Heart Study. N Engl J Med.

[12] Koren MJ, Devereux RB, Casale PN, Savage DD, Laragh JH (1991). Relation of left ventricular mass and geometry to morbidity and mortality in uncomplicated essential hypertension. Ann Intern Med.

[13] Gerdts E, Cramariuc D, de Simone G, Wachtell K, Dahlöf B, Devereux RB (2008). Impact of left ventricular geometry on prognosis in hypertensive patients with left ventricular hypertrophy (the LIFE study). Eur J Echocardiogr.

[14] Ganau A, Devereux RB, Roman MJ (1992). Patterns of left ventricular hypertrophy and geometric remodeling in essential hypertension. J Am Coll Cardiol.

[15] Oktay AA, Lavie CJ, Milani RV (2016). Current perspectives on left ventricular geometry in systemic hypertension. Prog Cardiovasc Dis.

[16] Muiesan ML, Salvetti M, Monteduro C (2004). Left ventricular concentric geometry during treatment adversely affects cardiovascular prognosis in hypertensive patients. Hypertension.

[17] Devereux RB, Roman MJ, Paranicas M (2000). Impact of diabetes on cardiac structure and function: the strong heart study. Circulation.

[18] Eguchi K, Kario K, Hoshide S, Ishikawa J, Morinari M, Shimada K (2005). Type 2 diabetes is associated with left ventricular concentric remodeling in hypertensive patients. Am J Hypertens.

[19] McDonagh TA, Metra M, Adamo M (2023). 2023 Focused Update of the 2021 ESC Guidelines for the diagnosis and treatment of acute and chronic heart failure. Eur Heart J.

[20] Lundin M, Heiberg E, Nordlund D (2023). Prognostic utility and characterization of left ventricular hypertrophy using global thickness. Sci Rep.

[21] Fan JH, Wang JB, Wang SM, Abnet CC, Qiao YL, Taylor PR (2018). Longitudinal change in blood pressure is associated with cardiovascular disease mortality in a Chinese cohort. Heart.

[22] Armstrong BG (1998). Effect of measurement error on epidemiological studies of environmental and occupational exposures. Occup Environ Med.

[23] Stokholm ZA, Bonde JP, Christensen KL, Hansen AM, Kolstad HA (2013). Occupational noise exposure and the risk of hypertension. Epidemiology.

[24] Nagin DS, Tremblay RE (2001). Analyzing developmental trajectories of distinct but related behaviors: a group-based method. Psychol Methods.

[25] Nagin DS, Odgers CL (2010). Group-based trajectory modeling in clinical research. Annu Rev Clin Psychol.

[26] Li W, Jin C, Vaidya A (2017). Blood pressure trajectories and the risk of intracerebral hemorrhage and cerebral infarction: a prospective study. Hypertension.

[27] Yuan Z, Yang Y, Wang C (2018). Trajectories of long-term normal fasting plasma glucose and risk of coronary heart disease: a prospective cohort study. J Am Heart Assoc.

[28] Chu C, Liao YY, He MJ (2022). Blood pressure trajectories from childhood to youth and arterial stiffness in adulthood: a 30-year longitudinal follow-up study. Front Cardiovasc Med.

[29] Sabbah HN, Ilsar I, Zaretsky A, Rastogi S, Wang M, Gupta RC (2011). Vagus nerve stimulation in experimental heart failure. Heart Fail Rev.

[30] Heusch G (2008). Heart rate in the pathophysiology of coronary blood flow and myocardial ischaemia: benefit from selective bradycardic agents. Br J Pharmacol.

[31] Morcet JF, Safar M, Thomas F, Guize L, Benetos A (1999). Associations between heart rate and other risk factors in a large French population. J Hypertens.

[32] Palatini P (2011). Role of elevated heart rate in the development of cardiovascular disease in hypertension. Hypertension.

[33] Zhang N, Pan Q, Yang S (2025). Utilizing circadian heart rate variability features and machine learning for estimating left ventricular ejection fraction levels in hypertensive patients: a composite multiscale entropy analysis. Biosensors (Basel).

[34] Chinese Guidelines for the Prevention and Treatment of Hypertension Revision Committee (2024). Chinese guidelines for the prevention and treatment of hypertension (2024 revised edition) [in Chinese]. Chin J Hypertens.

[35] Sagara K, Goto K, Maeda M, Murata F, Fukuda H (2024). Medication adherence and associated factors in newly diagnosed hypertensive patients in Japan: the LIFE study. J Hypertens.

[36] Wang J, Ma JJ, Liu J, Zeng DD, Song C, Cao Z (2017). Prevalence and risk factors of comorbidities among hypertensive patients in China. Int J Med Sci.

[37] Yip W, Wong TY, Jonas JB (2013). Prevalence, awareness, and control of hypertension among Asian Indians living in urban Singapore and rural India. J Hypertens.

[38] Cohn JN, Ferrari R, Sharpe N (2000). Cardiac remodeling—concepts and clinical implications: a consensus paper from an international forum on cardiac remodeling. J Am Coll Cardiol.

[39] Muller JE, Stone PH, Turi ZG (1985). Circadian variation in the frequency of onset of acute myocardial infarction. N Engl J Med.

[40] Kario K, Pickering TG, Umeda Y (2003). Morning surge in blood pressure as a predictor of silent and clinical cerebrovascular disease in elderly hypertensives: a prospective study. Circulation.

[41] Somers VK, Dyken ME, Mark AL, Abboud FM (1993). Sympathetic-nerve activity during sleep in normal subjects. N Engl J Med.

[42] Scheer F, Hilton MF, Mantzoros CS, Shea SA (2009). Adverse metabolic and cardiovascular consequences of circadian misalignment. Proc Natl Acad Sci U S A.

[43] Li J (2012). Introduction to the Self-Rating Scale of Sleep (SRSS) [in Chinese]. Chin J Health Psychol.

[44] (2016). Recommendations for cardiac chamber quantification by echocardiography in adults: an update from the American Society of Echocardiography and the European Association of, Cardiovascular Imaging. Eur Heart J Cardiovasc Imaging.

[45] Mancia G, Kreutz R, Brunström M (2023). 2023 ESH Guidelines for the management of arterial hypertension The Task Force for the management of arterial hypertension of the European Society of Hypertension: Endorsed by the International Society of Hypertension (ISH) and the European Renal Association (ERA). J Hypertens.

[46] Williams B, Mancia G, Spiering W (2018). 2018 ESC/ESH Guidelines for the management of arterial hypertension. Eur Heart J.

[47] Julius S, Kjeldsen SE, Weber M (2004). Outcomes in hypertensive patients at high cardiovascular risk treated with regimens based on valsartan or amlodipine: the VALUE randomised trial. Lancet.

[48] Muntner P, Shimbo D, Carey RM (2019). Measurement of blood pressure in humans: a scientific statement from the American Heart Association. Hypertension.

[49] Whelton PK, Carey RM, Aronow WS (2018). 2017 ACC/AHA/AAPA/ABC/ACPM/AGS/APhA/ASH/ASPC/NMA/PCNA guideline for the prevention, detection, evaluation, and management of high blood pressure in adults: executive summary: a report of the American College of Cardiology/American Heart Association task force on clinical practice guidelines. Hypertension.

[50] Shah MS, Brownlee M (2016). Molecular and cellular mechanisms of cardiovascular disorders in diabetes. Circ Res.

[51] Frangogiannis NG (2019). The extracellular matrix in ischemic and nonischemic heart failure. Circ Res.

[52] Nesti L, Pugliese NR, Sciuto P (2022). Effect of empagliflozin on left ventricular contractility and peak oxygen uptake in subjects with type 2 diabetes without HEART disease: results of the EMPA-HEART trial. Cardiovasc Diabetol.

[53] Zheng NS, Annis J, Master H (2024). Sleep patterns and risk of chronic disease as measured by long-term monitoring with commercial wearable devices in the All of Us Research Program. Nat Med.

[54] Martino TA, Oudit GY, Herzenberg AM (2008). Circadian rhythm disorganization produces profound cardiovascular and renal disease in hamsters. Am J Physiol Regul Integr Comp Physiol.

[55] Robinson BF (1967). Relation of heart rate and systolic blood pressure to the onset of pain in angina pectoris. Circulation.

[56] Inoue R, Ohkubo T, Kikuya M (2012). Predictive value for mortality of the double product at rest obtained by home blood pressure measurement: the Ohasama study. Am J Hypertens.

[57] Asayama K, Hozawa A, Taguri M (2017). Blood pressure, heart rate, and double product in a pooled cohort: the Japan Arteriosclerosis Longitudinal Study. J Hypertens.

[58] Vidal-Petiot E, Ford I, Greenlaw N (2016). Cardiovascular event rates and mortality according to achieved systolic and diastolic blood pressure in patients with stable coronary artery disease: an international cohort study. Lancet.

[59] Ascher SB, de Lemos JA, Lee M (2022). Intensive blood pressure lowering in patients with malignant left ventricular hypertrophy. J Am Coll Cardiol.

[60] Benza RL, Ghofrani HA, Grünig E (2021). Effect of riociguat on right ventricular function in patients with pulmonary arterial hypertension and chronic thromboembolic pulmonary hypertension. J Heart Lung Transplant.

[61] Allen NB, Khan SS (2021). Blood pressure trajectories across the life course. Am J Hypertens.

[62] Hao G, Wang X, Treiber FA, Harshfield G, Kapuku G, Su S (2017). Blood pressure trajectories from childhood to young adulthood associated with cardiovascular risk: results from the 23-year longitudinal Georgia stress and heart study. Hypertension.

[63] Eguchi K, Ishikawa J, Hoshide S (2007). Differential impact of left ventricular mass and relative wall thickness on cardiovascular prognosis in diabetic and nondiabetic hypertensive subjects. Am Heart J.

